# Nd:YAG Laser Hyaloidotomy: A Therapeutic Approach for Valsalva Premacular Hemorrhage

**DOI:** 10.7759/cureus.56872

**Published:** 2024-03-25

**Authors:** Miguel E Hernández-Emanuelli, Jose J Echegaray, Andres Emanuelli

**Affiliations:** 1 School of Medicine, Ponce Health Sciences University, Ponce, PRI; 2 Ophthalmology, University of Puerto Rico, Medical Sciences Campus, San Juan, PRI; 3 Ophthalmology, Retina Care, San Juan, PRI

**Keywords:** subhyaloid hemorrhage, sudden vision loss, laser hyaloidotomy, valsalva, nd:yag

## Abstract

Subhyaloid hemorrhage, characterized by localized vitreoretinal detachment due to blood accumulation, often results in sudden vision loss, especially in the macular area. This case report highlights a 23-year-old female presenting with exercise-related Valsalva retinopathy leading to premacular subhyaloid hemorrhage. The patient underwent neodymium-doped yttrium aluminum garnet (Nd:YAG) laser hyaloidotomy, a non-invasive procedure, leading to rapid blood drainage and visual recovery.

The patient’s initial visual acuity was severely impaired, with a significant premacular hemorrhage obscuring the macula. A week later, due to the expanding hemorrhage, Nd:YAG laser hyaloidotomy was performed, demonstrating successful blood dispersion and restoration of vision. Follow-up revealed significant improvement with demarcation of the previous hemorrhage and no evidence of new findings.

The case emphasizes the importance of prompt intervention and considers alternative treatments for premacular subhyaloid hemorrhage. While associated with ocular pathologies, such as macular holes and retinal detachment, Nd:YAG laser hyaloidotomy remains a safe and effective outpatient procedure for managing premacular subhyaloid hemorrhage, avoiding the risks of more invasive surgical interventions. The presented case highlights the significance of tailored interventions based on patient history, minimizing the need for invasive procedures and their associated risks.

## Introduction

Subhyaloid hemorrhage, defined as a localized detachment of the vitreous from the retina caused by blood accumulation, leads to a sudden and profound loss of vision when it occurs in the macular area [1,2]. It may manifest in various conditions, including retinal vascular disorders such as proliferative diabetic retinopathy, retinal vein occlusion, macroaneurysm, age-related macular degeneration, and arteriovenous communication of the retina [3]. Additionally, subhyaloid hemorrhage may arise in the context of hematological disorders such as aplastic anemia and leukemia, as well as following procedures such as laser in situ keratomileusis [3]. It can also occur after retinal vascular rupture associated with physical exertion (Valsalva) [3].

As described by Durukan et al., hemorrhagic retinopathy of Valsalva refers to the rupture of superficial capillaries caused by an increase in retinal venous pressure, typically resulting from a sudden change in intrathoracic or intra-abdominal pressure [4]. This rapid elevation in intraocular venous pressure may lead to spontaneous rupture in superficial retinal capillaries, resulting in a sudden and painless decrease in visual acuity in an otherwise healthy eye [4]. This increase in pressure can occur during activities such as forceful coughing, sneezing, vomiting, or heavy lifting [5]. Numerous involuntary movements may resemble an involuntary Valsalva maneuver in daily life, yet they typically have no clinical repercussions [5].

We report this case to highlight the role of neodymium-doped yttrium aluminum garnet (Nd:YAG) laser hyaloidotomy in the management of premacular subhyaloid hemorrhage leading to early visual recovery.

## Case presentation

**Figure 1:**
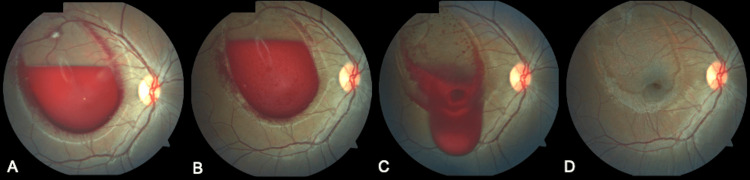
Color fundus photography. Color fundus photographs of a patient with Valsalva retinopathy. (A) Demonstrating boat-shaped hemorrhage. (B) One week later with persistent hemorrhage. (C) The same patient immediately after Nd:YAG laser hyaloidotomy. Note the draining premacular hemorrhage. (C) Fundus picture after treatment eight weeks later.

A 23-year-old Hispanic female arrived at our clinic for evaluation of sudden vision loss. The patient reported sudden-onset painless vision loss in the right eye with three days of evolution. She denied trauma and associated the sudden vision loss with her exercise training, which included weight lifting. Medical, family, and ocular history were unremarkable. The patient underwent a comprehensive review of systems and ophthalmological evaluation. On examination, the best-corrected visual acuity was hand motion and 20/20 on the right eye and left eye, respectively. Intraocular pressures were 17 mmHg in the right eye and 16 mmHg in the left eye. The anterior segment examination in both eyes was normal. A dilated fundus examination of the left eye was normal. The right eye revealed a subhyaloid hemorrhage obscuring the macula measuring six disc diameters (DD) extending beyond the superior arcade (Figure 1, Panel A). Hematological and clotting investigations were normal.

One week after the presentation, the subhyaloid hemorrhage increased by 0.5 DD (Figure 1, Panel B). Conservative treatment versus surgical treatment with potential complications was discussed. The retina specialist opted for using an Nd:YAG laser to create an opening in the hyaloid membrane, facilitating the dispersion of blood into the vitreous cavity for absorption. Mydriasis was accomplished with a 1.0% tropicamide eye drop and topical anesthesia with 0.5% proparacaine (Alcaine) eye drops. An Nd:YAG slit-lamp mounted laser using a Goldman three-mirror contact lens (Volk) delivered single bursts at 1.8 mJ to perforate the posterior hyaloid membrane. The laser beams were directed at the most prominent part of the premacular hemorrhage sparing the fovea and retinal blood vessels. Most of the blood quickly flowed into the inferior vitreous, restoring vision (Figure 1, Panel C).

Due to poor follow-up, the patient arrived eight weeks later. Her vision had improved to 20/25 in the right eye. On fundus examination, the left eye remained healthy. The right eye showed demarcation where the previous hemorrhage occurred, with no evidence of blood in the macular region or any new findings (Figure 1, Panel D). The patient was discharged, and a follow-up appointment was arranged six months later.

## Discussion

Premacular subhyaloid hemorrhage can lead to a sudden and significant loss of vision, which may persist if left untreated [1-4]. This type of hemorrhage is often recognized as a distinct, circular, or dumbbell-shaped accumulation of bright red blood either below the internal limiting membrane (ILM) or between the ILM and the hyaloid face, typically located in or around the macular region [1,2]. While spontaneous resolution commonly occurs, the duration can vary from weeks to months, influenced by the amount and thickness of the blood involved [6]. Its occurrence in both eyes or individuals with only one functional eye can severely impair vision. Additionally, it presents a risk of permanent visual impairment due to changes in macular pigmentation, the development of epiretinal membranes, and potential toxic damage to the retina resulting from prolonged exposure to hemoglobin and iron [3].

Several methods have been outlined for addressing premacular subhyaloid hemorrhage. These encompass observation, Nd:YAG laser hyaloidotomy, pneumatic displacement achieved through intravitreal gas injection, tissue plasminogen activator, and pars plana vitrectomy [3,7]. Perforating the posterior hyaloid face using Nd:YAG or green argon laser constitutes a non-invasive technique [3,7]. This method allows for the drainage of extensive premacular subhyaloid hemorrhage into the vitreous, promoting the absorption of blood cells. This, in turn, enhances vision within days by clearing the obstructed macular area [3,7]. However, the ultimate visual prognosis depends on the root cause of the subhyaloid hemorrhage and any concomitant retinal alterations [4].

This treatment method provides prompt intervention upon diagnosis, rapidly enhancing visual acuity [8,9]. It eliminates the necessity to subject the patient to risks and adverse events linked to blood stasis or early surgical intervention, such as pars plana vitrectomy, which carries risks such as cataract development and the potential for endophthalmitis, particularly in young patients like the one in this case [8-10]. However, using this laser is also associated with developing ocular pathologies, including macular holes, retinal detachment, and epiretinal membranes [8,9].

## Conclusions

In summary, Valsalva retinopathy is marked by sizable preretinal hemorrhages, and a thorough medical history in such cases can be pivotal for a more precise diagnosis. Spontaneous resolution can occur over time, but, in some cases, medical intervention may be necessary to address complications and aid in visual recovery. Nd:YAG laser hyaloidotomy stands out as an affordable, efficient, and safe outpatient procedure for treating premacular subhyaloid hemorrhage. It expeditiously drains blood, restoring visual function without requiring more invasive vitreoretinal procedures and their associated severe complications.
